# Children and adolescents’ views on coping with stress and aversive feelings – a thematic analysis

**DOI:** 10.1186/s12889-025-23878-8

**Published:** 2025-08-02

**Authors:** Siri Jakobsson Støre, Sven Hassler, Louise Persson, Linda Beckman

**Affiliations:** 1 School of Behavioural, Social and Legal Sciences, Örebro University, Örebro, SE-701 82 Sweden; 2 Department of Social and Psychological Studies, Karlstad University, Karlstad, Sweden; 3 Department of Health Sciences, Karlstad University, Karlstad, Sweden

**Keywords:** Adolescent, Child, Coping, Group interview, Mental health, Resilience

## Abstract

**Background:**

The study objective was to explore children and adolescents’ views on coping with stress and aversive feelings.

**Methods:**

Swedish children and adolescents from Värmland county, aged 10–14 (*N* = 44, girls = 26), participated in group interviews on health and coping. In total, eight group interviews were conducted at four different schools.

**Results:**

Data was analyzed with reflexive thematic analysis and resulted in three themes: *Accepting and expressing feelings*, *Doing things that make you feel good*, and *Searching for help*.

**Conclusions:**

Encouraging healthy emotional expression, support-seeking, and challenging societal norms can, according to the analysis of children and adolescents’ testimonies, foster resilience. Family, peers, student health services, professionals, teachers, and animals contribute to their coping resources, with trust and relatability playing significant roles. The implications of the study can inform and improve future child health interventions and educational programs for nurturing effective coping skills from an early age.

**Supplementary Information:**

The online version contains supplementary material available at 10.1186/s12889-025-23878-8.

## Background

Levels of stress and aversive feelings have increased among Nordic adolescents in recent years [1]. This increase primarily involves internalizing problems such as depression symptoms, anxiety, and stress-related issues, which can manifest in somatic complaints like pain and sleep difficulties [2]. Research highlights significant long-term consequences of these problems for adolescents. For instance, a longitudinal study found that somatic symptoms such as stomach aches and headaches during adolescence predicted mental health problems six years later, including clinical anxiety and depression [3]. Additionally, stress and emotional difficulties in adolescence have been associated with challenges in educational attainment, social relationships, and overall life satisfaction in adulthood [4]. Zooming in on Sweden, data from the Swedish Health Behavior in School-aged Children (HBSC) study revealed that 77% of 15-year-old girls and 46% of boys reported experiencing at least two subjective health complaints, such as stomach ache, headache, sleeping problems, or dizziness weekly [1]. Moreover, statistics from The Swedish Board of Health and Welfare indicated a troubling increase in rates of depression and self-harm among girls [5].

On the contrary, research also indicates that Swedish adolescents today generally report good mental well-being [6]. A suggested explanation for this discrepancy is that the same words might be used to describe both everyday problems and more severe problems [7]. This argument of mixing trivial with severe symptoms has, however, been contested by Högberg et al. analysis of trends in psychosomatic complaints in Swedish adolescents [8]. The authors argue that “binary categorisations of students can be reductive and conceal important variations across students” [8]. In light of the discrepancy between reports of general good mental well-being and reports of increased mental ill-health among children and adolescents, it is essential to investigate children and adolescents’ thoughts and perspectives on health matters, and how they talk about feelings, mental health problems, and strategies to cope with such problems [9].

Coping can be defined as individuals’ thoughts and behaviors to handle internal and external stressful situations [10]. Different theoretical coping models have been proposed, including problem-focused coping versus emotion-focused coping [11]. As de Boo and Wicherts write, “Coping is expected to moderate and mediate associations between stress and mental health problems” [10, 12].

Previous research on coping strategies among children and adolescents has primarily focused on how they handle identity and other developmental aspects [13]. Additionally, research has suggested that the context in which individuals are situated significantly affects their coping strategies [14]. A Swedish focus group interview study with adolescents (15–18 years old) explored their views on mental health and found that it was partly seen as dependent on the adolescents’ situation, thoughts and coping mechanisms [15]. The study emphasized the importance of supporting adolescents in expressing how they feel and in managing everyday life stressors. Given that coping strategies tend to vary across different developmental stages and often are situation-dependent, the current study aimed to explore children and adolescents’ views on coping with stress and aversive feelings in the context of their schools.

The study aimed to address the gap in research on children and adolescents’ views on coping with aversive feelings and stress. Despite the growing focus on promoting mental health among children, little is known about how they themselves perceive and articulate effective coping strategies. By exploring these perspectives, the study sought to contribute to a deeper understanding of the resources and relationships that support children and adolescents’ emotional resilience, with the goal of informing interventions and educational programs tailored to their needs.

## Method

### Design

This study employed a qualitative exploratory design which aimed to capture children and adolescents’ lived experiences and perspectives through semi-structured group interviews. Group interviews were selected to encourage dynamic discussions, enabling children and adolescents to build on one another’s responses and provide richer insights into their experiences [16]. The study design reflects an inductive and abductive orientation, prioritizing children and adolescents’ voices while contextualizing findings within existing knowledge The abductive approach facilitated the identification of patterns and themes that emerged from the data while remaining open to unexpected findings and new insights.

### Settings

Karlstad Municipality’s central student health services in Värmland, Sweden, launched a health education program in 2021 for 10–14-year-olds children and adolescents. The program was led by school nurses/counsellors and teachers in three 60-minute sessions at middle and junior high school levels. The sessions were adapted to each age group and covered (I) physical health: nutrition, activity, sleep, and balance; (II) psychosocial health: thoughts, stress management, and coping with worries and anxiety; and (III) conclusion: recap of the previous sessions, self-esteem boosting and mindfulness exercises. The interviews were conducted after the health education program. The program’s emphasis on psychosocial health and coping strategies created an opportunity to explore children and adolescents’ own views on coping with stress and aversive feelings.

### Sampling strategy

Recruitment was facilitated by the central student health services, who helped identify four schools. Children were invited to participate through announcements on an online school platform for parents. There were no specific selection criteria, and all children who expressed interest in participating were eligible.

### Participants

The study involved 44 children and adolescents (26 girls and 18 boys) in grades 4–8 (ages 10–14) (Table 1). Since we did not collect any personal information about the participants, the sample age mean and SD are unknown. All eight groups consisted of a mix of boys and girls, varying from four to eight children in each group. This range was chosen to balance the need for a manageable group dynamic with the aim of promoting active participation and discussions. Smaller groups (four to six participants) allow for more individual contributions and detailed reflections, while slightly larger groups (up to eight participants) might enhance the diversity of perspectives and interaction within the group [17]. All four schools were based in middle to high socioeconomic areas. The children new each other from school. Personal information such as ethnic background, religion, and sexual orientation was not collected. The names in the results section are pseudonyms, to protect confidentiality of the children and adolescents.

**Table 1:** Background characteristics of the participating children and adolescents (*N* = 44).

**Background characteristics**	**n (%)**
Gender	
Girls	26 (59.09)
>Boys	18 (40.91)
Grade	
4th grade	7 (15.90)
5th grade	14 (31.82)
6th grade	6 (13.64)
8th grade	17 (38.64)
School area	
North school	9 (20.45)
East school	17 (38.64)
South school	10 (22.73)
West school	8 (18.18)

### Procedure

The current study conducted semi-structured qualitative group interviews with children and adolescents to explore their perceptions about health [18], and coping with stress and aversive feelings. The interview guide, developed by the research group, was reviewed in advance by school nurses and school counsellors to ensure relevance and clarity. The primary questions for the current study were: “When you feel sad, down, stressed, anxious, what are some common ways you try to manage those feelings?” and “Are there specific activities, strategies, or people you turn to for support?”

To minimize the risk of any single participant – such as a particularly outspoken or popular student – dominating the conversation, the moderators were attentive to group dynamics throughout the interviews. We used open-ended questions and made sure to actively invite contributions from all participants. When necessary, we gently redirected the discussion to encourage quieter children to share their perspectives, to create a supportive and inclusive environment. Some of the questions required clarification when interviewing the youngest participants. We anticipated this and had prepared age-appropriate wording and follow-up prompts. During the interviews, we adapted the phrasing when necessary and used concrete examples to support understanding. We also checked in with the children to ensure they had understood the question before moving forward. These adjustments helped ensure that all participants could engage meaningfully in the discussion.

### Data collection

Eight group interviews took place at the children and adolescents’ schools between April 2022 and January 2023. Each interview lasted between 30.33 and 59.37 min and was recorded using a dictaphone. To facilitate dynamic discussions and encourage the exchange of ideas, group interviews were chosen as the research method. The interviews were conducted in pairs by the authors, with one serving as the moderator and the other as an observer. The distribution of participants per group interview is shown in Table 2.

**Table 2:** Distribution of participants by group and grade across school areas

School area	Group	Grade	Total
North school			9
	Group 1	Grade 8	4
	Group 2	Grade 8	5
East school			17
	Group 1	Grade 6	6
	Group 2	Grade 5	6
	Group 3	Grade 4	5
South school			10
	Group 1	Grade 5	6
	Group 2	Grade 4	2
		Grade 5	2
West school			8
	Group 1	Grade 8	8

### Data analysis

The audio files were transcribed verbatim, with slight alterations of quotations when translated from Swedish to English. Data were analyzed with reflexive thematic analysis according to Braun and Clarke, a widely utilized method employed to identify, analyze, interpret, and report recurring patterns in qualitative data [19]. Thematic analysis was chosen for several reasons. Firstly, thematic analysis is a highly flexible method that can be applied across different frameworks and research questions. This makes it an ideal choice for exploring children and adolescents’ perspectives on coping with stress and aversive feelings, as it allows for both an in-depth understanding of individual experiences and the identification of patterns across the group. This flexibility enables the method to adapt to the diverse nature of the data collected. Secondly, thematic analysis is well-suited to handle data from group interviews, enabling both individual and collective perspectives on coping strategies. Thirdly, the research questions were centered around how children and adolescents express and cope with aversive feelings and what resources support their coping mechanisms. Thematic analysis allowed us to systematically examine the data and organize it into meaningful themes that directly addressed these questions. The method provides the flexibility to focus on both the content of children and adolescents’ narratives and the underlying structures that influence their coping strategies.

The analytic process involved several distinct phases. Initially, data was thoroughly read and re-read to develop familiarity. Then, an analysis of the data was conducted to identify its core topics. Each new relevant topic was assigned a unique code. A systematic search for themes was undertaken in the subsequent phase, linking coded elements to preliminary themes. Author SJS conducted the coding, and the themes were collaboratively discussed until a consensus was reached. The identified themes underwent a thorough review before being named and defined in the subsequent phase. Lastly, a final analysis of selected extracts was conducted, and the report was written. The presented quotes in the results section were labeled with pseudonyms and school levels. The study followed the COREQ checklist for reporting group interviews [20], and the Reflexive Thematic Analysis Reporting Guidelines [21].

### Reflexivity

The research team consisted of two associate professors (SH, LB) and one assistant professor (LP) in public health sciences, as well as a PsyD clinical psychologist and assistant professor in psychology (SJS). One researcher identifies as a man (SH), and the rest of the team members identify as women (SJS, LP, LB). The team had diverse expertise in conducting qualitative interviews, particularly with children and adolescents, which provided a broad foundation for understanding their experiences of coping with stress and aversive feelings.

The varied disciplinary backgrounds of the researchers influenced the way in which the study was framed and analyzed. Public health scholars (LB, LP, SH) brought an understanding of health education, stress management, and the role of social contexts in coping strategies, while SJS, a clinical psychologist, provided expertise in child and adolescent mental health, particularly in relation to how children and adolescents manage emotional distress and aversive feelings. These perspectives were essential in guiding the study’s focus on how children and adolescents cope with stress and aversive feelings.

The researchers were mindful of their own experiences and potential biases, especially when interpreting how children and adolescents perceive and cope with stressful and aversive feelings. For instance, SJS’s clinical background may have led to a heightened sensitivity to psychological mechanisms involved in coping, while the public health scholars might have been more inclined to explore coping strategies in relation to environmental or social factors. These differing viewpoints were considered throughout the research process, ensuring that the findings were integrated and in line with the data.

### Ethical considerations

The study adhered to ethical guidelines throughout the research process, ensuring that participants’ rights and well-being were respected. The Swedish Ethical Review Authority approved the study (Dnr C2021/885). Children and their parents were provided with both oral and written information about the study, emphasizing that participation was voluntary and that they could withdraw at any time. Written consent was obtained from both children and their parents before proceeding with the interviews.

Given the sensitive nature of the topics discussed - stress, coping and aversive feelings - extra care was taken to foster a supportive and non-judgmental environment during the interviews. The fact that some children and adolescents were in the same classes could raise potential concerns related to confidentiality and the influence of peers being present during the interviews. To address this, it was clearly communicated to the children that the information would be treated confidentially by the research team, and that no information should leave the room.

An important ethical consideration involved the power dynamics between the researchers and the participants. The team recognized that the children and adolescents might perceive the researchers’ professional status as intimidating, which could influence their willingness to share personal experiences. To mitigate this, the research team emphasized the voluntary nature of participation and provided reassurances regarding confidentiality and anonymity. Pseudonyms were used in the reporting of results to further protect children and adolescents’ identities.

## Results

The thematic analysis resulted in 47 codes and three themes. Example codes from the first theme, *Accepting and expressing feelings*, include “Dare to be vulnerable” and “Write it down”. Example codes from the second theme, *Doing things that make you feel good*, include “Play games”, “Listen to music”, and “Distraction”. Finally, example codes from the third theme, *Searching for help*, include “Support family” and “Trust”. See Table S1 in the supplemental material for an overview of themes and associated codes. The themes, taken separately or together, aim to explore how children and adolescents view coping with stress and aversive feelings. There was a general agreement on individual differences and situation-dependent differences regarding how one copes with aversive feelings.

### Accepting and expressing feelings

The first theme was about accepting the situation and the aversive feelings associated with it, and/or expressing these feelings one way or another. Children and adolescents conveyed that it is important to acknowledge and accept feelings to be able to move on – feelings come and go, and it is okay not to feel great all the time. One participant saw this as being “the roller coaster of life”, i.e., that feeling aversive feelings is an inherent part of being human. Children and adolescents also expressed an acceptance of these feelings in terms of allowing them to come and go and not struggle with them, and had noticed that if you let feelings stay for a while, they will disappear by themselves. In stressful times, children and adolescents described that they tried to focus on their breathing. They also talked about the importance of having time alone for self-reflection.

There was also a clear sense of personal responsibility when it came to emotional expression. Many children and adolescents said they had come to understand the importance of taking the initiative to talk about their feelings, rather than waiting for others to notice:

It’s up to you to take responsibility. If you just hold everything inside, then you’re just going to feel worse. That’s why I feel like, okay, I have to do this; I have to express how I feel; otherwise, I’m just going to spiral downward if I keep it all to myself. So, I think that it’s your responsibility to break the barrier. (Celeste, grade 8)

This account reflects a nuanced awareness of how emotional suppression can escalate into greater distress over time, and how expressing oneself – even if uncomfortable – can serve as a form of emotional regulation. The metaphor of “breaking the barrier” also illustrates the inner resistance many people experience when trying to open up, and the courage it takes to push through that. At the same time, some participants emphasized more private ways of expressing their feelings, e.g., by writing about their sad experiences and feelings. They differentiated between short- and long-term consequences of expressing their feelings; for example, that it could feel uncomfortable to be vulnerable and talk about their feelings at the moment, but this was something that could make them feel better in the long run.

Children and adolescents also demonstrated insight into the complexity of emotions – particularly the distinction between what someone feels and what they show to the outside world. They were attuned to the fact that people often mask their inner experiences, sometimes for the sake of others or to avoid judgment:

It’s different from one person to the next. People can be really depressed and not let it show, they can seem happy and laugh all the time, but when they’re by themselves it’s a different thing, then they are really sad. Whereas other people show it all. (Aurora, grade 8)

This observation reflects a growing understanding of emotional nuance – how the emotional world can be hidden beneath outward appearances, and how people cope differently based on context, personality, and support systems. Participants also recognized that even the same person might switch between strategies depending on how safe or supportive the environment felt, such as being more expressive at home but reserved at school.

Furthermore, some described emotions as deeply personal, and not always something that should be shared with other people. Moreover, children and adolescents also enclosed that they did not necessarily want to burden others with their feelings. They tended to compare their problems to other people’s problems, which could affect their choices of coping strategies. This balancing of degree of distress also involved expressions of critical self-reflection and self-doubts, such as “maybe I’m exaggerating”. Gender norms also played a significant role in shaping how emotions were expressed. Many participants reflected on how societal expectations influence whether and how boys and girls show vulnerability. The common phrase “boys don’t cry” was brought up as an example of these norms, with some acknowledging that boys might feel just as deeply, but often internalize their distress:

It’s more common to see a girl being vulnerable and sad than it is to see a boy in that way. But in reality, there’s no difference. Of course, boys can be equally sad, I just think they hold it inside more. (Parker, grade 8)

### Doing things that make you feel good

The second theme was about different ways children and adolescents distracted themselves to feel better in stressful times. Many of them described engaging in various activities that served as a buffer against emotional distress—consciously or unconsciously redirecting their attention away from difficult thoughts and feelings. This redirection was not necessarily about avoiding problems completely, but rather about carving out temporary moments of relief. For some, this meant doing things that they associated with comfort, joy, or a sense of normalcy. Disclosures about deliberate acts of shifting focus from the stressor to something more manageable or pleasurable were common, and often spoken about in a matter-of-fact tone, suggesting that these strategies had become a familiar part of their emotional coping repertoire:

It’s important to do things that make you feel good, that push away the stress and all that. So that’s what I try to do when I’m feeling stressed. (Leo, grade 8)

This quote captured the core idea that small, enjoyable acts could serve as anchors in the midst of emotional turmoil. This drive to “push away the stress” was echoed by many, who spoke about a range of go-to activities that helped them regulate their emotional states. Other examples of such activities were Lego building, drawing, beadwork, audiobooks, and engaging in physical exercise – practices that not only distracted but also provided a sense of immersion, focus and sometimes even mastery. Entertainment in the form of playing games, listening to music, and watching films were also frequently mentioned, offering an escape from unwanted feelings or intrusive thoughts:

I like to exercise because then you think about something else and not about what happened. I don’t know whether it’s true or not, but I read in the newspaper that endorphins are released in the body when you exercise, which make you happy. (Mika, grade 6).

This statement suggests a developing awareness of the mind-body connection and a belief in the biological mechanisms behind emotional relief, reflecting how some children and adolescents combine personal experience with information they’ve picked up from the media or school.

In addition to solitary activities, socializing with peers and parents to temporarily forget their problems was a common coping mechanism. Being with others was described as a way to shift the emotional focus outward, either by sharing laughter and conversation or by simply being in a familiar and comforting presence. This could take different forms depending on the setting and emotional need:

If it happens when I’m at school, I try to be with as many people as possible because then you start to talk about other things, which make you feel better. If I’m at home, I try to be by myself for a while, and then I go to my parents and do an activity with them. (Kenai, grade 5)

Children and adolescents expressed awareness about the fact that distraction is not an optimal long-term solution for resolving problems but more a tool for managing acute stress and for regaining a sense of stability and control before having to “deal with it later”, when in a calmer state of mind. The theme also includes disclosures of denial in terms of not acknowledging the existence or severity of the aversive feelings, and of suppressing the problem – consciously pushing away thoughts and feelings. Children and adolescents expressed that feelings are difficult to talk about and that it does not always feel like talking about them helps, at least not in the short run.

### Searching for help

One common coping strategy shared by children and adolescents was seeking support from trusted individuals when facing emotional distress or overwhelming situations. These disclosures revealed how central interpersonal relationships are to the regulation of stress, and how the presence of a supportive figure can significantly alter one’s ability to cope. Many participants spoke of turning to their family members – particularly their parents – for emotional support. Parents were often portrayed as safe and dependable, people who not only listened but also provided comfort and perspective. The act of opening up usually took place during familiar and emotionally available moments, such as at bedtime or after a challenging day, and these small rituals of connection seemed to hold particular emotional significance:

When my mom comes to my room to say good night, I usually tell her what I’m thinking about, and then she always comes up with a good answer. (Freddie, grade 4)

This account highlights how even brief, everyday interactions can become important opportunities for emotional disclosure and reassurance. These moments created space for children to make sense of their thoughts, while also receiving calm and nurturing responses from someone they trusted. The emphasis on the parent as someone who “always comes up with a good answer” illustrates the value placed on emotional competence and wisdom in caregivers. However, these supportive dynamics were not universal. Some children and adolescents expressed hesitation or discomfort about confiding in their parents, fearing that their concerns might trigger excessive worry or unwanted intervention. There was a sense among some participants that once something was shared, it might spiral into actions they could not control – such as referrals to professionals or school staff – especially if the parent misunderstood the severity or intention behind their disclosure. In these instances, friends emerged as valuable confidants. Friendships were seen as reciprocal and emotionally attuned, with peers often being viewed as more relatable and less likely to overreact.

The choice of whom to turn to often depended on the nature of the problem. For issues related to academic stress or school-related anxiety, children and adolescents identified teachers as accessible sources of help. Teachers were described not only as authority figures but as emotionally present adults who could offer immediate relief through practical support – such as adjusting deadlines or offering time off from certain tasks:

If you feel stressed out at school, you can talk to your teacher and get some adjustments to your schoolwork that day, so you can breathe and rest for a while. (René, grade 6).

This statement points to an emerging self-advocacy among students, where seeking adjustments is recognized as a legitimate coping mechanism, not a sign of weakness. It also underscores the role of teachers as first-line responders in managing academic stress. While student health services were sometimes mentioned, teachers were often preferred due to the existing relationships and daily contact. Nevertheless, concerns about confidentiality were present – particularly among those who feared their disclosures might be passed on to parents or other adults without their consent, compromising their sense of safety and autonomy.

Beyond human relationships, children and adolescents also found emotional comfort in their pets and other animals. These animals were described as calming, nonjudgmental companions that could be trusted with secrets. Talking to animals was viewed as a way to process feelings, brainstorm solutions, and feel a sense of connection, which helped alleviate stress. One participant reflected on the relaxing effects of spending time with a rabbit:

It’s relaxing to just sit and pet it. Then you just think about the rabbit and it’s so calming to me. (Abel, grade 5)

These words reflect how the physical act of petting an animal can anchor a person in the present moment, shifting attention away from distressing thoughts and into a more peaceful mental state. The rabbit becomes more than a pet – it becomes a tool for self-soothing and emotional containment.

Gender differences were also described within this theme, particularly regarding norms and expectations around emotional expression. Boys, in particular, noted that seeking help or showing vulnerability could be socially risky. Some described internalized messages suggesting that expressing sadness or anxiety might lead to mockery or loss of social status, especially among male peers. This led to a tendency among some boys to suppress their emotions or delay help-seeking until things became unmanageable. Finally, several participants emphasized that seeking help was not just about receiving support – it was also about offering it to others. Children and adolescents described a strong sense of moral responsibility when it came to their friends’ wellbeing. Even in situations where they were sworn to secrecy, many felt compelled to intervene if they believed their friend was in serious distress. The decision to involve an adult – despite the risk of damaging the friendship – was framed as a courageous and necessary act:

If it has gone too far, you - as their friend - have to tell their parents about it and say, “Your child does not feel well, your child needs help.” (Jannick, grade 8).

## Discussion

The study aimed to explore children and adolescents’ views on coping with stress and aversive feelings. Semi-structured qualitative group interviews were conducted including questions on health and coping. Data were analyzed with reflexive thematic analysis, and resulted in three themes: (I) *Accepting and expressing feelings*, (II) *Doing things that make you feel good*, and (III) *Searching for help*. The first theme, *Accepting and expressing feelings*, included disclosures about emotion-focused coping, which centers on managing and regulating emotional responses, i.e., active steps to alleviate aversive feelings rather than passively avoiding them [10]. Focusing on breathing was mentioned, indicating an awareness of the mind-body connection and the potential to influence emotional states through physiological processes. Breathing to foster mindfulness has also been the core focus of another school-based health intervention for adolescents [22]. A strong sense of self-responsibility in utilizing active coping strategies was also a visible feature of the narratives, resonating with Eriksson’s study on adolescents’ mental preparedness and sense of self-responsibility when it comes to crises and emergencies [23].

Writing and verbalizing feelings and challenges as a way to counteract the detrimental effects of internalizing feelings was mentioned by the children and adolescents as a means of processing their feelings. This aligns with established therapeutic techniques, such as journaling, which encourage emotional expression and self-reflection [24]. Furthermore, children and adolescents’ recognition of short-term discomfort for long-term emotional well-being suggests a nuanced understanding of the dynamic interplay between immediate emotional responses and lasting mental health outcomes. The narratives revealed a duality in their perceptions of social engagement and solitude. While interactions with friends and engaging in activities were seen as beneficial, they also acknowledged the value of spending time alone for self-reflection. This duality points to the importance of a balanced approach to coping, where both social environment resources and individual resources contribute to emotional resilience [25]. They also exhibited a high level of maturity by emphasizing the importance of acknowledging and accepting feelings. This perspective challenges what is perhaps a societal norm of constantly pursuing positive feelings and highlights the children and adolescents’ understanding that emotional diversity is an inherent aspect of the human experience.

The second theme, *Doing things that make you feel good*, is in line with distraction strategies in Ayers et al. coping model, when facing aversive feelings and stressors [26]. Distraction entails intentionally diverting attention from distressing feelings, thoughts, or situations towards engaging and absorbing activities for temporary relief from emotional distress [10]. Engaging in activities that foster absorption and engagement allowed them to shift their focus away from negative feelings, contributing to a break in the cycle of rumination and emotional escalation. Leisure and recreational activities also served as outlets for relaxation as well. Social interaction was another powerful distraction strategy. Conversations with peers and parents were identified as a means to redirect attention from internal turmoil. The children and adolescents articulated an understanding that while distraction can provide immediate relief, it is not a method for resolving the underlying issues causing stress. This reflects a mature perspective, indicating that children and adolescents are aware of the limitations of distraction as a coping mechanism and its role in providing temporary stability. This is also in line with de Boo and Wicherts, who concluded that pro-social children could shift between different, and many coping strategies [10]. Most people are able to use a wide range of coping strategies but perhaps less flexible when feeling stressed [27].

The theme also included disclosures bordering on avoidance strategies employed by children and adolescents in response to stress and aversive feelings. These strategies encompass a range of behaviors aimed at evading confrontation with the stressor [10]. Denial, where the existence or severity of the problem is not acknowledged, and suppression, which involves consciously pushing away thoughts and feelings, were examples of strategies employed. This inclination to keep feelings hidden was motivated by a desire to protect loved ones from additional burdens. The apprehension of burdening others reflects a level of empathy and consideration among children and adolescents, revealing their sensitivity to the emotional well-being of those around them (perhaps sometimes at their own expense). Children and adolescents also engaged in a comparative evaluation of their stressors with those of others. They often downplayed their distress, considering it less significant than the distress of others. This evaluation led them to minimize their feelings and avoid seeking help, even when it might be beneficial. A notable aspect of the discussions was the influence of gender on emotional expression. Children and adolescents recognized societal gender norms and expectations, where boys are discouraged from showing vulnerability or sadness. This gendered perspective demonstrates children and adolescents’ awareness of cultural norms surrounding emotions. However, they also emphasized the shared emotional experiences between genders, and that feelings are fundamentally similar regardless of gender – suggesting that these gender differences in frequency and intensity of feelings are learned and that they vary across societies [28].

The third theme, *Searching for help*, centered around children and adolescents’ support-seeking strategies in response to stress and aversive feelings. Family members were a central source of support for children and adolescents in times of distress. The willingness of parents to listen, provide comfort, and offer advice created an environment of trust and safety that contributed to the children’s reliance on family support. The narratives underscored that certain individuals, such as parents and siblings, could be trusted with personal feelings and secrets, indicating that not everyone can be trusted. Previous research has shown that adolescents with access to adults who listen to them experience less anxiety about the future, suggesting that support-seeking strategies are effective [29].

In addition to family, peers and teachers played significant roles in children and adolescents’ support networks. Friends were often sought out as confidants, particularly due to their relatability and shared experiences. However, the quality of support provided by friends could vary, as peers might not always possess the skills to handle their own feelings. Teachers were also identified as potential sources of support, especially during the school day. However, there was also a recognition that teachers might not always be equipped to provide the emotional support needed, highlighting the importance of having a range of supportive figures.

The narratives revealed the unique role of animals in children and adolescents’ support networks, in line with previous research [30]. Animals, whether household pets or other animals, were viewed as nonjudgmental listeners and calming influences. Children and adolescents expressed a sense of trust and connection with animals, often seeking solace and comfort in their presence. Proximity to animals is associated with reduced physiological arousal and enhanced coping, for instance, when it comes to animal-assisted therapies in hospitalized children [31]. Gender dynamics played a significant role in this theme as well, particularly the perception that boys are discouraged from showing vulnerability, something that has been described in the literature for a long time [32]. This awareness influenced whom they felt comfortable seeking support from where girls were often described as more understanding and supportive. Interestingly, children and adolescents acknowledged their role in supporting their peers and that it could have a positive impact on their well-being. This recognition of empathy speaks to the development of prosocial behavior and highlights children’s growing sense of responsibility toward the emotional well-being of others. Empathy-related responding is also the main focus of certain school-based interventions [33].

Previous studies on coping in adolescents have emphasized interpersonal relationships as the most commonly cited sources of everyday stress [34]. However, research also highlights school as a significant source of stress, particularly related to academic demands, social dynamics, and performance pressure [1, 35]. Findings from the current study underscore school-related stress as a predominant concern among the children and adolescents. This emphasis may be attributed to the slightly younger age group in the present study (10–14 years) and the fact that the interviews were conducted within a school context. Additionally, regional variations in educational practices, the implementation of preventive programs, and cultural attitudes toward education could play a role.

The findings align with previous research indicating that active coping strategies tend to increase as children progress from middle school to junior high school, reflecting a developmental trend across adolescence [34, 36]. Studies have further emphasized the importance of promoting early knowledge and reducing stigma to support the identification of challenges and encourage help-seeking behaviors or adaptive coping strategies [37, 38]. These insights provide additional support for health education programs, such as the one implemented prior to the group interviews in this study.

The context of this study is a school-based health intervention, which is integral to the design and interpretation of the study, as it may have influenced the perspectives shared by children and adolescents. The timing of the interviews likely captured reflections that were shaped by the proximity to the intervention, but it may also limit insights into longer-term impacts. Previous research highlights that school-based interventions can play a critical role in supporting children and adolescent mental health, particularly when implemented early and tailored to the developmental needs of the age group [39]. However, evidence regarding the sustained effects of such programs remains mixed, with some studies indicating a decline in impact over time [40].

### Limitations

A first limitation is the recruitment process which may have favored more talkative and articulate children, potentially at the expense of other important perspectives. Secondly, due to privacy concerns and the small size of the participating schools, we chose not to collect detailed background information on children and adolescents to protect their anonymity. While we acknowledge the importance of such data for understanding how background factors, such as socio-economic status, might influence responses, the risk of identification in small school populations - especially when involving children - led us to exclude this information. Nonetheless, this is a limitation of the study as the results may not be transferable to children and adolescents in general. Future studies should aim to collect and incorporate more comprehensive background data to assess the extent to which individual differences contribute to the observed outcomes. Such data would also allow for a more nuanced discussion of how participant characteristics intersect with the study’s core themes.

A third limitation is that the study used group interviews to encourage discussion and interaction, as the mixed-gender groups potentially could have influenced the dynamic of the discussions, in addition to other unknown factors such as school class history and dynamics. A fourth limitation is that several extracts were difficult to code, as the same behavior (e.g., being together with another person) could either entail an active support-seeking strategy (confide feelings in someone – Accepting and expressing feelings), or passive avoidance (socializing to forget – *Doing things that make you feel good*), when the intention was not clearly stated. On the same note, not wanting to share feelings with others was most often coded under the passive distraction theme (*Doing things that make you feel good*) when it potentially entailed active self-support. Future qualitative interview studies should include more follow-up questions on the intentions behind different coping behaviors.

### Strengths

There is a general lack of research studies where children and adolescents have the opportunity to share their thoughts and perspectives. A strength of the current study is, therefore that it gave the participating children and adolescents a voice and the opportunity to express their perspectives on health and coping. According to Hermann et al., obtaining more knowledge about how children view health and coping, as well as their experiences with preventive interventions, can contribute to improve such interventions [15]. A second strength is that the authors have diverse backgrounds in psychology, public health, and social science, potentially enriching data interpretation. All four researchers were involved in the discussion of the thematic structure, which enhanced the trustworthiness of the study. Last but not least, an important strength of the current study is that it builds upon previously conducted studies in Sweden [15, 41], and that it contributes to new perspectives on coping, i.e., from younger children, in another region of the country. Värmland, like other regions, has unique social, cultural, and environmental factors that influence children and adolescents’ experiences and coping mechanisms.

### Implications

The findings of this study offer important considerations for the development of future interventions and support structures aimed at promoting emotional well-being in children and adolescents. While this study does not evaluate specific programs or provide evidence for long-term effects, the children and adolescents’ own perspectives shed light on key elements that they find helpful in managing stress and aversive feelings. Participants emphasized the importance of emotional openness, support-seeking, and access to both private and social coping strategies. These insights suggest that school environments may benefit from integrating opportunities for emotional reflection and support into daily routines – whether through classroom discussions, supportive teacher-student interactions, or designated calm spaces. Rather than proposing specific program models, our findings point to the value of trust, relatability, and flexibility in supporting children’s emotional coping.

Although some participants mentioned practices such as breathing exercises or writing, these should not be interpreted as endorsements of specific interventions. Rather, they highlight that certain techniques resonate with children when they feel safe and in control. Similarly, the central roles played by parents and teachers in the children’s coping narratives suggest that equipping adults with greater emotional literacy may strengthen the everyday support network available to young people. However, as noted in previous evaluations [2], educational programs vary widely in quality and impact. Therefore, future efforts should prioritize evidence-based approaches and include ongoing evaluation. Given the regional and contextual nature of the current study, these findings can also inform local initiatives in Värmland, Sweden. Listening to children’s own accounts of coping may guide schools and municipalities in tailoring their support to the specific needs and experiences of the children they serve. In sum, while the current study cannot speak to the long-term outcomes of particular interventions, it does emphasize the value of involving children and adolescents in the conversation about emotional health – both in shaping responsive environments and in informing future research on effective support strategies.

## Conclusions

The findings of this thematic analysis contribute to the growing body of literature on children and adolescents’ coping strategies and emotional well-being. Adolescents can use a variety of coping strategies to manage stress and aversive feelings. Their narratives reflect not only a repertoire of coping strategies but also a nuanced understanding of emotional dynamics, individual and situational differences, and the importance of self-responsibility. This understanding aligns with the complexity of human emotional experiences and underscores the need for personalized approaches to coping interventions. Children and adolescents’ awareness of the limitations of distraction as a coping strategy demonstrated their emotional maturity and emphasize the importance of a comprehensive coping toolkit that encompasses both immediate and sustainable strategies. Support from the family, peers, student health services, and professionals – along with, to a lesser extent, animals and teachers – contributed to children and adolescents’ coping toolkits. Trust, relatability, and the role of societal norms in shaping support-seeking behaviors are key takeaways in this regard. In conclusion, all of these insights have implications for the development of interventions and educational programs that can foster effective and nuanced coping skills from an early age, promoting emotional well-being throughout the lifespan.

## Supplementary Information

Supplementary Material 1.

## Data Availability

The data that support the findings of this study are available on reasonable request from the corresponding author.

## Declarations

The research was conducted in line with the Declaration of Helsinki. Both participants and their parents provided written informed consent to take part in the study.
