# Supplementary material for: Children and adolescents’ views on coping with stress and aversive feelings – a thematic analysis
Source: BMC Public Health. 2025 Aug 2;25:2625. doi: 10.1186/s12889-025-23878-8 (PMC12317445; doi:10.1186/s12889-025-23878-8)
Supplement: Supplementary file 1 — Supplementary Material 1. [file 12889_2025_23878_MOESM1_ESM.docx]

**Table S1**

*Overview of themes and associated codes*

| **Theme and codes** | **Frequency of codes** |
| --- | --- |
| Accepting and expressing feelings |  |
| Accept feelings | 5 |
| Balance | 2 |
| Boys don’t cry | 27 |
| Compare problems with others | 6 |
| Dare to be vulnerable | 2 |
| Focus on one task at a time | 2 |
| Individual differences | 4 |
| Motivation | 1 |
| Recognize feelings in others | 3 |
| Responsibility oneself | 10 |
| Self-support | 7 |
| Take deep breaths | 4 |
| Talk about it | 3 |
| Write it down | 1 |
|  |  |
| Doing things that make you feel good |  |
| Avoid stress | 1 |
| Difficult to talk about | 2 |
| Distraction | 11 |
| Drawing | 1 |
| Eat | 1 |
| Fight or flight | 1 |
| Have fun | 4 |
| Integrity | 2 |
| Lego building | 1 |
| Less mobile phone | 4 |
| Listen to music | 1 |
| Mobile phone and social media | 5 |
| Play games | 2 |
| Read | 4 |
| Rest and sleep | 3 |
| Sing and dance | 4 |
| Socialize | 1 |
| Sports and exercise | 7 |
|  |  |
| Searching for help |  |
| Age difference | 1 |
| Be with someone | 2 |
| Gender difference | 2 |
| Hide feelings | 8 |
| Not bring others down | 1 |
| Responsibility others | 6 |
| Situation dependent | 2 |
| Social media | 3 |
| Support animals | 9 |
| Support family | 26 |
| Support friend | 14 |
| Support professional | 6 |
| Support student health | 13 |
| Support teacher | 3 |
| Trust | 8 |
